# Plasma gelsolin levels and outcomes after aneurysmal subarachnoid hemorrhage

**DOI:** 10.1186/cc12828

**Published:** 2013-07-23

**Authors:** Jian-Wei Pan, Ling-Na He, Feng Xiao, Jian Shen, Ren-Ya Zhan

**Affiliations:** 1 Department of Neurosurgery, The First Affiliated Hospital, School of Medicine, Zhejiang University, 79 Qingchun Road, Hangzhou 310000, PR China; 2 College of Computer Science, Zhejiang University of Technology, 38 Zheda Road, Hangzhou 310000, PR China

**Keywords:** Gelsolin, Aneurysmal subarachnoid hemorrhage, Functional outcome, Mortality

## Abstract

**Introduction:**

Lower gelsolin levels have been associated with the severity and poor outcome of critical illness. Nevertheless, their link with clinical outcomes of aneurysmal subarachnoid hemorrhage is unknown. Therefore, we aimed to investigate the relationship between plasma gelsolin levels and clinical outcomes in patients with aneurysmal subarachnoid hemorrhage.

**Methods:**

A total of 262 consecutive patients and 150 healthy subjects were included. Plasma gelsolin levels were measured by enzyme-linked immunosorbent assay. Mortality and poor long-term outcome (Glasgow Outcome Scale score of 1-3) at 6 months were recorded.

**Results:**

Plasma gelsolin levels on admission were substantially lower in patients than in healthy controls (66.9 (26.4) mg/L *vs*. 126.4 (35.4) mg/L, *P *< 0.001), and negatively associated with World Federation of Neurological Surgeons score (*r *= -0.554, *P *< 0.001) and Fisher score (*r *= -0.538, *P *< 0.001), and identified as an independent predictor of poor functional outcome (odds ratio, 0.957; 95% confidence interval (CI), 0.933-0.983; *P *= 0.001) and death (odds ratio, 0.953; 95% CI, 0.917-0.990; *P *= 0.003) after 6 months. The areas under the ROC curve of gelsolin for functional outcome and mortality were similar to those of World Federation of Neurological Surgeons score and Fisher score (all *P *> 0.05). Gelsolin improved the predictive values of World Federation of Neurological Surgeons score and Fisher score for functional outcome (both *P *< 0.05), but not for mortality (both *P *> 0.05).

**Conclusions:**

Gelsolin levels are a useful, complementary tool to predict functional outcome and mortality 6 months after aneurysmal subarachnoid hemorrhage.

## Introduction

Plasma gelsolin is the extracellular isoform of a ubiquitous cytoplasmic actin-binding protein, gelsolin, which mediates cell shape changes and motility [1]. Plasma gelsolin is associated with the severity and outcome of critical illness, and therefore, has been proposed as a prognostic marker in acute illness [2-11]. Recently, it has been reported that plasma gelsolin levels were also decreased in the patients with traumatic brain injury [12], ischemic stroke [13], and intracerebral hemorrhage [14]; in these groups of patients, low gelsolin levels were highly predictive for mortality. Gelsolin has been also found to be depleted in the peripheral blood of patients with aneurysmal subarachnoid hemorrhage (SAH) [15]. However, no published information exists to date about the correlation of gelsolin with outcome after aneurysmal SAH. The present study aimed to investigate this correlation and to analyze the usefulness of gelsolin as a predictor of long-term functional outcome and mortality in aneurysmal SAH.

## Materials and methods

### Study population

Between June 2008 and August 2011, all patients with aneurysmal SAH confirmed by computed tomography (CT) angiography with or without digital subtraction angiography who were admitted to Department of Neurosurgery, The First Affiliated Hospital, School of Medicine, Zhejiang University were evaluated in the study. Inclusion criteria were clinical history of SAH within the last 24 h before admission and the treatment by surgery or coiling within 48 h after admission. Exclusion criteria were rebleeding after admission, aged <18 years, existing previous head trauma, neurological disease, use of antiplatelet or anticoagulant medication, presence of other prior systemic diseases including uremia, liver cirrhosis, malignancy, chronic heart or lung disease, diabetes mellitus, and hypertension. Healthy individuals were evaluated as controls if they presented to our hospital and had blood collected as part of medical examination in May 2011. The study was conducted in accordance with the guidelines approved by the Human Research Ethics Committee at The First Affiliated Hospital, School of Medicine, Zhejiang University. Written informed consent was obtained from the study subjects or their relatives.

### Clinical and radiological assessment

At admission, clinical severity was assessed using the World Federation of Neurological Surgeons (WFNS) score as follows: 1 = Glasgow Coma Scale (GCS) score of 15, no motor deficit; 2 = GCS score of 13-14, no motor deficit; 3 = GCS score of 13-14 and any motor deficit or aphasia; 4 = GCS score of 7-12, with or without motor deficit; and 5 = GCS of 3 to 6, with or without motor deficit [16]. The initial CT was classified according to the modified Fisher score as follows: grade 1 = no subarachnoid blood; grade 2 = broad diffusion of subarachnoid blood; grade 3 = with clots or thick layers of subarachnoid blood; grade 4 = intraventricular hemorrhage or intracerebral hematoma, no clot; and grade 5 = intraventricular hemorrhage or intracerebral hematoma with clot [17]. All CT scans were performed according to the neuroradiology department protocol. Investigators who read them were blinded to clinical information.

### Patient management

The type of treatment (surgery or coiling) was decided according to both location and size of the aneurysm by the neurosurgeon and the neuroradiologist. All patients received intravenous Nimodipine at a dose of 2 mg/h from admission until at least day 14, except during periods of uncontrolled increased intracranial pressure during which intravenous Nimodipine was discontinued. Seizures were systematically prevented by Sodium Valproate (200 mg × 3, per os). After surgery or coiling, patients were managed with 'triple H' therapy (hypertension with a mean arterial pressure goal >100 mm Hg, hypervolemia, and hemodilution with a goal hematocrit of 30) through 12 days after hemorrhage. An external ventricular drain was inserted in case of hydrocephalus on CT and in patients with a high WFNS grade (WFNS score of 3 to 5). Increased intracranial pressure was treated by cerebrospinal fluid drainage, mechanical ventilation, reinforcement of sedation, and, rarely, moderate hypothermia. CT was performed whenever clinical deterioration occurred to search for secondary complications such as hydrocephalus or ischemia.

Clinical onset of cerebral vasospasm was defined as the acute onset of a focal neurologic deficit or a change in the GCS score of 2 or more points. All suspected cases of cerebral vasospasms were confirmed by CT angiography and were then taken to the interventional radiology suite for cerebral angiography. Each vasospasm episode was treated with intra-arterial administration of Nimodipine as recently described. This therapy was repeated if necessary. Balloon angioplasty was used as a second-line therapy when Nimodipine was judged insufficient.

### Determination of gelsolin in plasma

The informed consents were obtained from study population or family members in all cases before the blood were collected. Venous blood in the healthy individuals or the patients was drawn at study entry or on admission. The blood samples were immediately placed into sterile EDTA test tubes and centrifuged at 1,500 g for 20 min at 4°C to collect plasma. Plasma was stored at -70°C until assayed. The concentration of gelsolin in plasma was analyzed by enzyme-linked immunosorbent assay (ELISA) using commercial kits (CoTimes, Beijing, China) in accordance with the manufactures' instructions. Intra-assay and inter-assay coefficients of variation were 4.2% and 6.8%, respectively. The blood samples were run in duplicate. Researchers running ELISAs were blinded to all patient details.

### Endpoint

Participants were followed up until death or completion of 6 months after SAH. The endpoints were unfavorable outcome and death after 6 months. The functional outcome was defined by Glasgow outcome scale (GOS) score. GOS was defined as follows: 1 = death; 2 = persistent vegetative state; 3 = severe disability; 4 = moderate disability; and 5 = good recovery [18]. GOS scores were dichotomized in favorable and unfavorable outcomes (GOS of 4-5 *vs*. GOS of 1-3). For follow-up, structured telephone interviews were performed by one doctor who was blinded to clinical information and gelsolin levels.

### Statistical analysis

Statistical analysis was performed with SPSS 10.0 (SPSS Inc., Chicago, IL, USA) and MedCalc 9.6.4.0. (MedCalc Software, Mariakerke, Belgium). The normality of data distribution was assessed by the Kolmogorovor-Smirnov test or Shapiro-Wilk test. All values are expressed as median (interquartile range), mean ± standard deviation or counts (percentage) unless otherwise specified. Comparisons were made by using: (1) chi-square test or Fisher exact test for categorical data; (2) unpaired Student *t *test for continuous normally distributed variables; and (3) the Mann-Whitney U-test for continuous non-normally distributed variables. Correlations of gelsolin with other variables were assessed by Spearman's correlation coefficient. The relations of gelsolin to the poor functional outcome (GOS 1-3) and death were assessed in a logistic-regression model. For multivariate analysis, we included the significantly different outcome predictors as assessed in univariate analysis. A ROC curve was configured to establish the cutoff point of plasma gelsolin with the optimal sensitivity and specificity for predicting the poor functional outcome (GOS 1-3) and mortality. A *P *value < 0.05 was considered statistically significant.

## Results

### Study population characteristics

During the recruitment period, 308 patients were admitted with an initial diagnosis of aneurysmal SAH, 269 (87.3%) patients fulfilled the inclusion criteria and exclusion criteria, and adequate data on admission and follow-up were available for 262 individuals (112 men and 150 women) (85.1%) who were finally included in the analysis. Table 1 summarized the other demographic, clinical, laboratory and radiological data from baseline CT scans of the patients. A control group consisted of 150 healthy individuals. The intergroup differences of patients and healthy controls in the age and sex were not statistically significant (both *P *> 0.05).

**Table 1:** The characteristics in patients with aneurysmal subarachnoid hemorrhage.

	All patients (*n *= 262)
Sex (male/female)	112/150
Age (years)	43.6 ± 12.1
WFNS score on admission	2 (2)
Fisher score on admission	2 (1)
Aneurysmal location	
Posterior communication artery	78 (29.8%)
Internal carotid artery	40 (15.3%)
Anterior communication artery	59 (22.5%)
Middle cerebral artery	39 (14.9%)
Anterior cerebral artery	30 (11.5%)
Posterior cerebral artery	10 (3.8%)
Vertebral artery	6 (2.3%)
Surgery	163 (62.2%)
Acute hydrocephalus	79 (30.2%)
Intraventricular hemorrhage	55 (21.0%)
External ventricular drain	91 (34.7%)
Vasospasm	112 (42.8%)
CT ischemia	39 (14.9%)
Admission time (h)	4.0 (4.1)
Plasma-sampling time (h)	5.6 (6.4)
Seizure	37 (14.1%)
Systolic arterial pressure (mmHg)	130 (31)
Diastolic arterial pressure (mmHg)	80 (22)
Mean arterial pressure (mmHg)	98 (24)
Heart rate (beats/min)	80 (35)
Body temperature (°C)	36.5 (0.9)
Respiratory rate (respirations/min)	18 (4)
Blood oxygen saturation (%)	88 (5)
Blood white blood cell count (×10^9^/L)	7.5 (4.2)
Blood hemoglobin level (g/L)	125 (38)
Blood platelet count (×10^9^/L)	174 (52)
Blood glucose level (mmol/L)	9.2 (3.0)
Blood sodium level (mmol/L)	136 (7)
Blood potassium level (mmol/L)	3.7 (0.9)
Prothrombin time (s)	14.6 (4.0)
Thrombin time (s)	18.9 ± 3.2
Partial thromboplastin time (s)	39.4 (10.8)
Plasma C-reactive protein level (mg/L)	6.4 (3.9)
Plasma fibrinogen level (g/L)	3.9 (4.1)
Plasma D-dimer level (mg/L)	1.8 (1.1)
Plasma gelsolin level (mg/L)	66.9 (26.4)

Numerical variables were presented as median (interquartile range) or mean ± standard deviation. Categorical variables were expressed as counts (percentage). Numerical variables were analyzed by Mann-Whitney U-test or unpaired Student *t *test. Categorical variables were analyzed by chi-square test or Fisher exact test.

*n*, number of patients; WFNS, World Federation of Neurological Surgeons.

### The change of plasma gelsolin level in SAH patients

**Figure 1:**
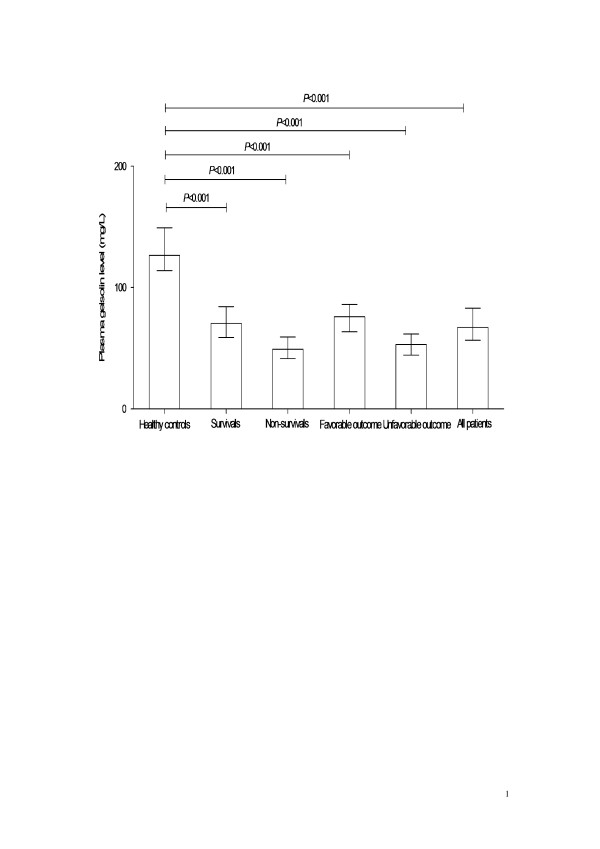
**The change of plasma gelsolin concentration in patients with aneurysmal subarachnoid hemorrhage**. Using Mann-Whitney U-test, the admission gelsolin levels were significantly decreased in all patients, survivals, non-survivals, patients with favorable outcome, and those with unfavorable outcome compared with healthy control individuals. Data are expressed as median (interquartile range).

Plasma gelsolin values are continuous non-normally distributed variables. Therefore, all values are expressed as median (interquartile range). Using the Mann-Whitney U-test, the admission gelsolin levels were significantly decreased in all patients (66.9 (26.4) mg/L), survivals (70.3 (25.5) mg/L), non-survivals (49.0 (17.9) mg/L), patients with favorable outcome (75.7 (22.6) mg/L), and those with unfavorable outcome (52.8 (17.5) mg/L) compared with healthy control individuals (126.4 (35.4) mg/L, all *P *< 0.001) (Figure 1).

### Correlations of plasma gelsolin level with disease severity

**Figure 2:**
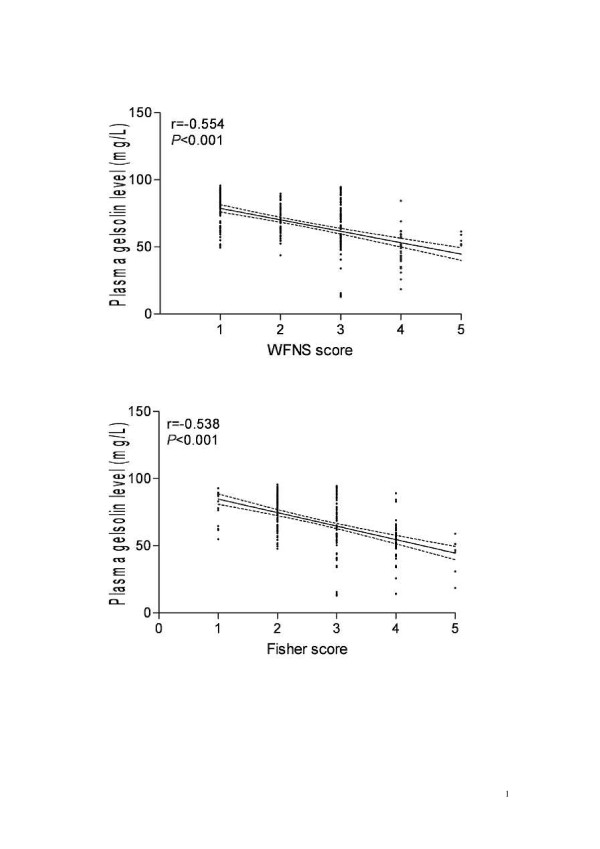
**The correlations of plasma gelsolin level with disease severity in patients with aneurysmal subarachnoid hemorrhage**. Plasma gelsolin level was highly associated with WFNS score and Fisher score using Spearman's correlation coefficient. WFNS indicates World Federation of Neurological Surgeons.

In all 262 patients with aneurysmal SAH, a significant correlation emerged between WFNS scores and plasma gelsolin level (r=-0.554, *P *< 0.001), as well as between Fisher scores and plasma gelsolin level (r=-0.538, *P *< 0.001) using Spearman's correlation coefficient (Figure 2).

### Poor neurologic function prediction

Seventy-three patients (27.9%) suffered from poor neurologic outcome (GOS 1-3) in 6 months. Table 2 shows that there was a significantly higher likelihood of poor neurologic outcome at 6 months in patients that have higher WFNS score, Fisher score, glucose, C-reactive protein, fibrinogen and D-dimer concentration, and lower gelsolin level, and that more frequently showed intraventricular hemorrhage, external ventricular drain, vasospasm, and CT ischemia. When the above variables found to be significant in the univariate analysis were introduced into the logistic model, a multivariate analyses selected WFNS score (odds ratio, 7.947; 95% confidence interval (CI), 1.398-16.210; *P *= 0.004), Fisher score (odds ratio, 9.772; 95% CI, 2.278-21.012; *P *= 0.002), and plasma gelsolin level (odds ratio, 0.957; 95% CI, 0.933-0.983; *P *= 0.001) as the independent predictors for 6-month poor neurologic outcome of patients.

**Table 2:** The factors associated with 6-month function outcome in patients with aneurysmal subarachnoid hemorrhage.

	GOS 1-3 (*n *= 73)	GOS 4-5 (*n *= 189)	Univariate analysis *P *value
Sex (male/female)	34/39	78/111	0.463
Age (years)	44.5 ± 10.5	43.2 ± 12.7	0.847
WFNS score on admission	3 (1)	2 (2)	< 0.001
Fisher score on admission	4 (1)	2 (1)	< 0.001
Aneurysmal location			0.761
Posterior communication artery	27 (37.0%)	51 (27.0%)	
Internal carotid artery	11 (15.1%)	29(15.3%)	
Anterior communication artery	15 (20.5%)	44 (23.3%)	
Middle cerebral artery	10 (13.7%)	29 (15.3%)	
Anterior cerebral artery	6 (8.2%)	24 (12.7%)	
Posterior cerebral artery	2 (2.7%)	8 (4.2%)	
Vertebral artery	2 (2.7%)	4 (2.1%)	
Surgery	46 (63.0%)	117 (61.9%)	0.868
Acute hydrocephalus	39 (53.4%)	40 (21.2%)	< 0.001
Intraventricular hemorrhage	44 (60.3%)	11 (5.8%)	< 0.001
External ventricular drain	51 (69.9%)	40 (21.2%)	< 0.001
Vasospasm	56 (76.7%)	56 (29.6%)	< 0.001
CT ischemia	20 (27.4%)	19 (10.1%)	< 0.001
Admission time (h)	4.0 (3.5)	4.0 (4.7)	0.259
Plasma-sampling time (h)	5.4 (5.6)	5.6 (6.6)	0.204
Seizure	14 (19.2%)	23 (12.2%)	0.144
Systolic arterial pressure (mmHg)	129 (34)	131 (30)	0.748
Diastolic arterial pressure (mmHg)	79 (18)	81 (24)	0.706
Mean arterial pressure (mmHg)	98 (20)	97 (25)	0.530
Heart rate (beats/min)	81 (34)	79 (35)	0.716
Body temperature (°C)	36.4 (1.6)	36.5 (0.9)	0.381
Respiratory rate (respirations/min)	19 (3)	18 (4)	0.150
Blood oxygen saturation (%)	88 (5)	89 (5)	0.795
Blood white blood cell count (×10^9^/L)	7.8 (5.8)	7.4 (3.7)	0.607
Blood hemoglobin level (g/L)	124 (36)	125 (39)	0.409
Blood platelet count (×10^9^/L)	173 (43)	174 (56)	0.863
Blood glucose level (mmol/L)	10.4 (4.6)	8.9 (2.4)	< 0.001
Blood sodium level (mmol/L)	136 (8)	136 (8)	0.916
Blood potassium level (mmol/L)	3.7 (1.0)	3.7 (0.9)	0.904
Prothrombin time (s)	14.2 (4.0)	14.8 (4.1)	0.601
Thrombin time (s)	18.8 ± 3.2	18.9 ± 3.2	0.942
Partial thromboplastin time (s)	40.3 (11.2)	39.3 (10.8)	0.332
Plasma C-reactive protein level (mg/L)	8.0 (5.9)	6.1 (3.3)	< 0.001
Plasma fibrinogen level (g/L)	5.3 (4.4)	3.7 (3.2)	0.001
Plasma D-dimer level (mg/L)	2.1 (1.6)	1.7 (1.0)	0.003
Plasma gelsolin level (mg/L)	52.8 (17.5)	75.7 (22.6)	< 0.001

Numerical variables were presented as median (interquartile range) or mean ± standard deviation. Categorical variables were expressed as counts (percentage). Numerical variables were analyzed by Mann-Whitney U-test or unpaired Student *t *test. Categorical variables were analyzed by chi-square test or Fisher exact test.

GOS, Glasgow outcome scale; *n*, number of patients; WFNS, World Federation of Neurological Surgeons.

**Figure 3:**
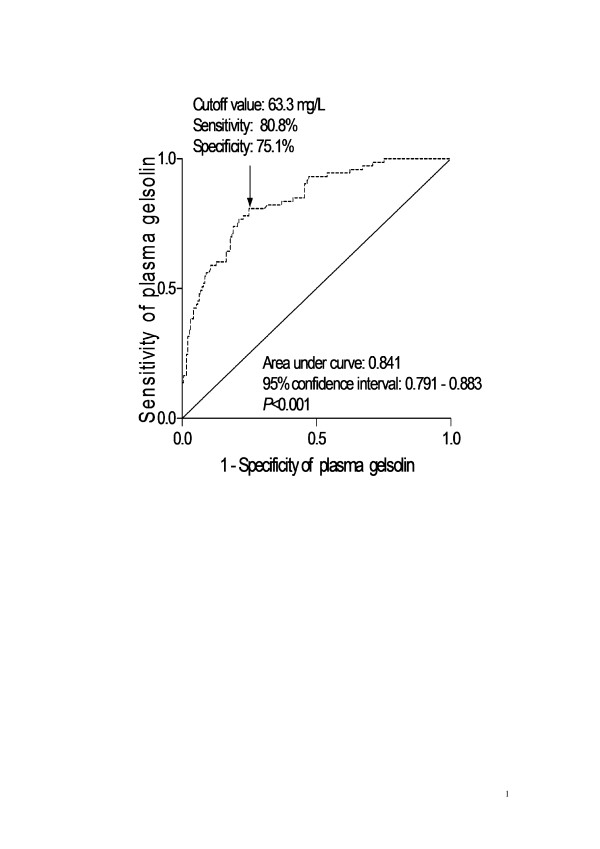
**ROC curve analysis of plasma gelsolin concentration for identifying patients with 6-month unfavorable outcome**. ROC curves were constructed based on the sensitivity and specificity of the plasma gelsolin concentration for identifying 6-month unfavorable outcome. The AUC was calculated based on the ROC curves and expressed as 95% CI. AUC ranges from 0.5 to 1.0. An AUC closer to 1 indicates a higher predictive power.

A ROC curve identified that a baseline plasma gelsolin level < 63.3 mg/L predicted 6-month poor neurologic outcome of patients with 80.8% sensitivity and 75.1% specificity (area under curve (AUC), 0.841; 95% CI, 0.791-0.883) (Figure 3). The predictive value of the gelsolin concentration was thus similar to those of WFNS score (AUC, 0.894; 95% CI, 0.850-0.929) (*P *= 0.091) and Fisher score (AUC, 0.886; 95% CI, 0.841-0.922) (*P *= 0.170). In a combined logistic-regression model, gelsolin improved the AUC of WFNS score to 0.930 (95% CI, 0.892 - 0.957) (*P *= 0.016) and the AUC of Fisher score to 0.923 (95% CI, 0.884 - 0.952) (*P *= 0.032).

### Mortality prediction

Thirty-one patients (11.8%) died from SAH in 6 months. Table 3 shows that there was a significantly higher likelihood of death at 6 months in patients that have higher WFNS score, Fisher score, glucose, C-reactive protein, fibrinogen and D-dimer concentration, and lower gelsolin level, and that more frequently showed intraventricular hemorrhage, external ventricular drain, vasospasm and CT ischemia. When the above variables found to be significant in the univariate analysis were introduced into the logistic model, a multivariate analyses selected WFNS score (odds ratio, 8.582; 95% CI, 2.028-18.484; *P *= 0.004), Fisher score (odds ratio, 9.594; 95% CI, 2.842-22.384; *P *= 0.003), and plasma gelsolin level (odds ratio, 0.953; 95% CI, 0.917-0.990; *P *= 0.003) as the independent predictors for 6-month mortality of patients.

**Table 3:** The factors associated with 6-month mortality in patients with aneurysmal subarachnoid hemorrhage.

	Non-survival group (*n *= 31)	Survival group (*n *= 231)	Univariate analysis *P *value
Sex (male/female)	13/18	99/132	0.922
Age (years)	44.4 ± 12.0	43.5 ± 12.2	0.695
WFNS score on admission	4 (1)	2 (2)	< 0.001
Fisher score on admission	4 (1)	2 (1)	< 0.001
Aneurysmal location			0.974
Posterior communication artery	7 (22.6%)	71 (30.7%)	
Internal carotid artery	6 (19.4%)	34 (14.7%)	
Anterior communication artery	7 (22.6%)	52 (22.5%)	
Middle cerebral artery	5 (16.1%)	34 (14.7%)	
Anterior cerebral artery	4 (12.9%)	26 (11.3%)	
Posterior cerebral artery	1 (3.2%)	9 (3.9%)	
Vertebral artery	1 (3.2%)	5 (2.2%)	
Surgery	20 (64.5%)	143 (61.9%)	0.778
Acute hydrocephalus	20 (64.5%)	59 (34.2%)	< 0.001
Intraventricular hemorrhage	27 (87.1%)	28 (12.1%)	< 0.001
External ventricular drain	28 (90.3%)	63 (27.3%)	< 0.001
Vasospasm	28 (90.3%)	84 (36.4%)	< 0.001
CT ischemia	12 (38.7%)	27 (11.7%)	< 0.001
Admission time (h)	4.0 (6.0)	4.0 (4.0)	0.826.
Plasma-sampling time (h)	6.0 (7.4)	5.4 (6.4)	0.398
Seizure	7 (22.5%)	30 (13.0%)	0.174
Systolic arterial pressure (mmHg)	130 (36)	130 (30)	0.373
Diastolic arterial pressure (mmHg)	81 (21)	80 (22)	0.421
Mean arterial pressure (mmHg)	101 (23)	97 (25)	0.257
Heart rate (beats/min)	81 (23)	80 (36)	0.602
Body temperature (°C)	36.7 (1.9)	36.5 (0.9)	0.551
Respiratory rate (respirations/min)	19 (3)	18 (5)	0.935
Blood oxygen saturation (%)	88 (7)	88 (5)	0.344
Blood white blood cell count (×10^9^/L)	6.5 (6.1)	7.5 (4.0)	0.566
Blood hemoglobin level (g/L)	122 (37)	125 (37)	0.129
Blood platelet count (×10^9^/L)	183 (34)	173 (56)	0.269
Blood glucose level (mmol/L)	10.7 (5.9)	9.1 (2.6)	0.003
Blood sodium level (mmol/L)	135 (11)	136 (7)	0.673
Blood potassium level (mmol/L)	3.8 (1.3)	3.7 (0.9)	0.926
Prothrombin time (s)	14.5 (3.2)	14.6 (4.1)	0.659
Thrombin time (s)	18.4 ± 2.9	18.9 ± 3.3	0.369
Partial thromboplastin time (s)	39.5 (11.8)	39.4 (10.6)	0.476
Plasma C-reactive protein level (mg/L)	8.1 (6.5)	6.2 (3.6)	0.013
Plasma fibrinogen level (g/L)	4.8 (4.2)	3.9 (3.8)	0.036
Plasma D-dimer level (mg/L)	2.3 (1.2)	1.8 (1.1)	0.014
Plasma gelsolin level (mg/L)	49.0 (17.9)	70.3 (25.5)	< 0.001

Numerical variables were presented as median (interquartile range) or mean ± standard deviation. Categorical variables were expressed as counts (percentage). Numerical variables were analyzed by Mann-Whitney U-test or unpaired Student *t *test. Categorical variables were analyzed by chi-square test or Fisher exact test.

*n*, number of patients; WFNS, World Federation of Neurological Surgeons.

**Figure 4:**
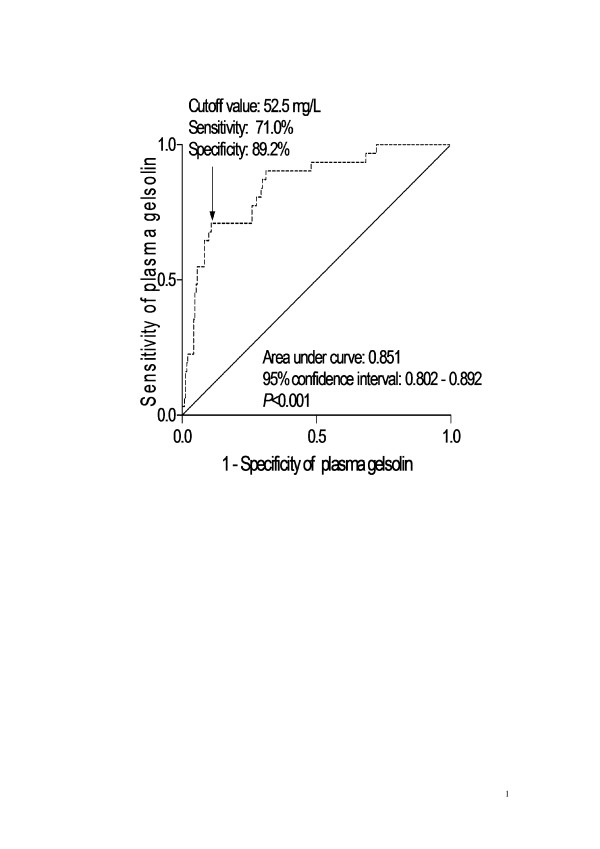
**ROC curve analysis of plasma gelsolin concentration for identifying patients with 6-month mortality**. ROC curves were constructed based on the sensitivity and specificity of the plasma gelsolin concentration for identifying 6-month mortality. The AUC was calculated based on the ROC curves and expressed as 95% CI. AUC ranges from 0.5 to 1.0. An AUC closer to 1 indicates a higher predictive power.

A ROC curve identified that a baseline plasma gelsolin level < 52.5 mg/L predicted 6-month mortality of patients with 71.0% sensitivity and 89.2% specificity (AUC, 0.851; 95% CI, 0.802-0.892) (Figure 4). The predictive value of the gelsolin concentration was thus similar to those of WFNS score (AUC, 0.910; 95% CI, 0.869-0.942) (*P *= 0.146) and Fisher score (AUC, 0.927; 95% CI, 0.889-0.956) (*P *= 0.053). In a combined logistic-regression model, gelsolin improved the AUC of WFNS score to 0.938 (95% CI, 0.902-0.964), and improved the AUC of Fisher score to 0.950 (95% CI, 0.916-0.973), but the differences were not significant (*P *= 0.141 and 0.227).

## Discussion

In this post hoc analysis of prospective collected data, we demonstrated that plasma gelsolin levels on admission in the patients were significantly lower than those in healthy controls; and in patients who had poor functional outcome or died in 6 months, the gelsolin levels on admission were significantly lower compared with levels in survivors or those with good functional outcome. In multivariate logistic regression models of predictors of death and poor functional outcome, the gelsolin levels on admission were an independent predictor.

Gelsolin is an 80-kDa actin-binding protein and is the first and most widely expressed member of a family of actin-severing proteins, which includes adseverin and villin [19]. After activation by micromolar Ca^2+^, gelsolin severs actin filaments, reducing actin cytoskeleton integrity, and remains bound to the barbed ends, inhibiting actin filament extension [20]. Upon reduction in free Ca^2+ ^to less than micromolar levels, and in the presence of polyphosphoinositides, gelsolin is released from the barbed ends, providing sites for rapid actin filament extension [20]. Thus, gelsolin appears to serve a critical role in actin filament dynamics [21-24]. Based on its actin-binding property, plasma gelsolin has been categorized as part of the extracellular 'actin-scavenging' system that counteracts actin toxicity when actin is released into the extracellular space [25]. Accordingly, the degree of plasma gelsolin depletion should reflect the degree of tissue injury that may lead to significant exposure of actin to the extracellular spaces. In every acute tissue injury setting examined, including toxic, hyperoxic, and idiopathic lung injury, adult respiratory distress syndrome, acute liver injury, myonecrosis, pancreatitis, trauma, burns and bacterial and protozoal sepsis, plasma gelsolin levels are subnormal [2-11]. The lower the levels of plasma gelsolin, the less favorable the prognosis of acute illness becomes [8-11]. Overall, gelsolin is associated with the severity and outcome of critical illness, and therefore, has been proposed as a prognostic marker in acute illness. Previous studies have shown significant predictive values of gelsolin in other acute brain injury including intracerebral hemorrhage, ischemic stroke, and traumatic brain injury [12-14]. This study showed that gelsolin was closely related to WFNS and Fisher scores, which in turn were associated with outcome of SAH in a multivariate regression model. Furthermore, gelsolin was identified as a reliable and independent marker to predict 6-month outcome in patients with SAH. Importantly, gelsolin's discriminative power (reflected by AUC) was in the range of WFNS and Fisher scores which are known to be a strong individual outcome predictor. Gelsolin improved AUC of WFNS and Fisher scores for functional outcome. However, gelsolin could not improve AUC of WFNS and Fisher scores for mortality. Overall, our data suggest that plasma gelsolin level in this early period might reflect the initial insult (as demonstrated by the close relation between gelsolin and WFNS score or Fisher scores) and gelsolin might have an interesting potential as a new prognostic biomarker.

The mechanism of plasma gelsolin's actions is poorly understood. Gelsolin was first discovered as an intracellular protein involved in actin dynamics [26]. Plasma gelsolin was subsequently identified as a secreted isoform of the cytoplasmic gelsolin [27]. Similar to cytoplasmic gelsolin, plasma gelsolin also severs and scavenges actin [28-30], a major body protein that may be exposed or released by cellular injury and that may enhance some major components of proinflammatory cytokine production, and impair the microcirculation and compromise multiple organs [31,32]. In addition to actin, plasma gelsolin binds and modulates bioactive lipids, such as endotoxin [33], lysophosphatidic acid [34], and platelet activating factor [35]. This effect may partially explain how exogenous gelsolin replacement significantly enhances survival of septic animals [8], and blunts the inflammatory response in animal models of lung injury [36] and burns [7]. Based on these data, we propose that plasma gelsolin functions as an important endogenous guard against overwhelming inflammation from tissue injuries.

Gelsolin is constitutively expressed throughout the central nervous system and is particularly concentrated in neuronal growth cones [37]. It has been demonstrated that gelsolin modulates voltage-dependent Ca^2+ ^channels and N-methyl-D-aspartate receptor-coupled channel activity and reduces vulnerability to excitotoxicity in cultured hippocampal neurons after its activation by Ca^2+ ^[38] and that gelsolin ^-/- ^mice are more susceptible to brain injury after ischemia/reperfusion and gelsolin could serve as a neuroprotective factor in murine cerebral ischemia [39]. Recent results suggested that enhanced gelsolin expression is an important mechanism by which histone deacetylase inhibitor trichostatin A protects against ischemic brain injury [40]. A recent paper reported plasma gelsolin is decreased and correlated with rate of decline in Alzheimer's disease [41]. From the available information, it is suggested that reversing plasma gelsolin deficiency may be an effective treatment for SAH.

In addition, univariate analysis showed that some parameters including blood glucose level, plasma C-reactive protein level, plasma fibrinogen level, and plasma D-dimer level were associated with 6-month poor functional outcome and mortality. But, a multivariate logistic regression did not verify these results. These differences may be caused by sample size or study design. However, this can supply some information about potentials of these biomarkers such as blood glucose level, plasma C-reactive protein level, plasma fibrinogen level, and plasma D-dimer level as outcome predictors in aneurysmal SAH.

## Conclusions

In this study, gelsolin levels are associated with clinical severity and are a useful, complementary tool to predict functional outcome and mortality 6 months after aneurysmal subarachnoid hemorrhage.

## Key messages

- In the patients with aneurysmal subarachnoid hemorrhage, plasma gelsolin level on admission was substantially lower than that in healthy controls.

- Plasma gelsolin level was highly negatively associated with World Federation of Neurological Surgeons score and Fisher score after aneurysmal subarachnoid hemorrhage.

- Plasma gelsolin level was an independent predictor of poor long-term functional outcome and death 6 months after aneurysmal subarachnoid hemorrhage.

- Plasma gelsolin level was a useful, complementary tool to predict poor long-term functional outcome and mortality 6 months after aneurysmal subarachnoid hemorrhage.

## Abbreviations

CT: computed tomography; ELISA: enzyme-linked immunosorbent assay; GCS: Glasgow Coma Scale; GOS: Glasgow outcome scale; SAH: subarachnoid hemorrhage; WFNS: World Federation of Neurological Surgeons.

## Competing interests

The authors declare that they have no competing interests.

## Authors' contributions

JWP and LNH contributed to the design of the study and drafted the manuscript and participated in the laboratory work. FX and JS enrolled the patients and contributed to data analysis and interpretation of the results. RYZ contributed to data analysis and interpretation of the results. All authors read and approved the final manuscript.
